# Applying cusum-based methods for the detection of outbreaks of Ross River virus disease in Western Australia

**DOI:** 10.1186/1472-6947-8-37

**Published:** 2008-08-13

**Authors:** Rochelle E Watkins, Serryn Eagleson, Bert Veenendaal, Graeme Wright, Aileen J Plant

**Affiliations:** 1 Australian Biosecurity CRC, Faculty of Health Sciences, Curtin University of Technology, Perth, Australia; 2 Department of Spatial Sciences, Curtin University of Technology, Perth, Australia

## Abstract

**Background:**

The automated monitoring of routinely collected disease surveillance data has the potential to ensure that important changes in disease incidence are promptly recognised. However, few studies have established whether the signals produced by automated monitoring methods correspond with events considered by epidemiologists to be of public health importance. This study investigates the correspondence between retrospective epidemiological evaluation of notifications of Ross River virus (RRv) disease in Western Australia, and the signals produced by two cumulative sum (cusum)-based automated monitoring methods.

**Methods:**

RRv disease case notification data between 1991 and 2004 were assessed retrospectively by two experienced epidemiologists, and the timing of identified outbreaks was compared with signals generated from two different types of cusum-based automated monitoring algorithms; the three Early Aberration Reporting System (EARS) cusum algorithms (C1, C2 and C3), and a negative binomial cusum.

**Results:**

We found the negative binomial cusum to have a significantly greater area under the receiver operator characteristic curve when compared with the EARS algorithms, suggesting that the negative binomial cusum has a greater level of agreement with epidemiological opinion than the EARS algorithms with respect to the existence of outbreaks of RRv disease, particularly at low false alarm rates. However, the performance of individual EARS and negative binomial cusum algorithms were not significantly different when timeliness was also incorporated into the area under the curve analyses.

**Conclusion:**

Our retrospective analysis of historical data suggests that, compared with the EARS algorithms, the negative binomial cusum provides greater sensitivity for the detection of outbreaks of RRv disease at low false alarm levels, and decreased timeliness early in the outbreak period. Prospective studies are required to investigate the potential usefulness of these algorithms in practice.

## Background

Increasingly, automated monitoring methods are being applied to routinely collected population health data to facilitate the recognition of significant changes in the health indicators under surveillance. The implementation of automated monitoring methods has been associated with improved awareness of trends in health-related data, improved data sharing and integration, and an improved ability to detect and respond to health events [1].

Statistical process control methods such as cumulative sums (cusums) are among the most commonly used methods to monitor population health surveillance data. Cusums are powerful, yet reasonably straightforward to design and implement, and are considered well-suited to the task of detecting changes in surveillance data early [2-4]. Using cusums to monitor disease data traditionally assumes that the parameters which adequately describe the observed outcome of the disease processes when it is at an ideal level can be specified [5]. In practice, specifying parameters which adequately describe the process under surveillance can be difficult, particularly when the process and systems associated with data generation do not appear to be stable, and disturbances can vary in size and area. Over time, changes in disease surveillance methods can also produce apparent changes in disease incidence when no real change has occurred.

One alternative to using fixed parameters to describe the ideal behaviour of the process under surveillance is to use historical observations to estimate the parameters to be employed in monitoring [5]. This approach has been used in the cusum-based monitoring methods developed as part of the Early Aberration Reporting System (EARS) [6]. The EARS cusum-based aberration detection algorithms use recently observed data to inform the expected level of the data under surveillance. As such, these algorithms signal change from the recent past rather than from theoretically derived parameters or ideal values.

Determining an appropriate baseline period from which to derive parameters for automated monitoring is complex, as changes in surveillance methods and trends associated with changing disease epidemiology can limit the usefulness of long series of historical data for the identification of ideal process levels. For example, longer baselines can be useful as a mechanism to assist in identifying or down-weighting the influence of previous outbreaks on summary statistics; however, for some diseases, seasonal outbreaks may produce elevated baseline estimates over many months. There is little specific information available to guide the selection of appropriate baselines in individual public health surveillance applications.

Routine national notifiable disease surveillance methods in Australia do not yet include the use of automated algorithms for outbreak detection; however, the EARS algorithms (C1, C2 and C3) have been implemented at a national level in New Zealand for the surveillance of notifiable disease data [7]. The EARS algorithms provide early detection of real and simulated disease outbreaks [8], and these cusum methods, which require little baseline data, have been found to perform as well as methods that require greater amounts of historical data for baseline estimation [9]. Although the EARS C1, C2 and C3 algorithms have been found to detect outbreaks of public health interest, including the start of the influenza season [9], little is known about how the signalling pattern of these algorithms corresponds to the identification of events of public health interest among epidemiologists.

In contrast to the industrial applications for which cusums were originally designed [2], the use of cusums to monitor population health data provides additional challenges associated with monitoring the complex and variable process of disease transmission and detection. Cusums applied to public health surveillance data which have high variance have been associated with a greater than expected number of false alarms in comparison to data with low variance [10]. Similarly, public health surveillance data with fluctuating variance has also been associated with variable specificity of surveillance algorithms [11]. Although a negative binomial cusum may provide a means to moderate the reported high false alarm rates associated with the use of established cusum based on other statistical models, the performance of negative binomial cusums has not been widely investigated. This analysis aimed to investigate the correspondence between retrospective epidemiological evaluation of notifications of RRv disease in Western Australia, and the signals produced by two cusum-based automated monitoring methods; the widely used EARS C1, C2 and C3 cusums, and a negative binomial cusum.

## Methods

The performance of the EARS C1, C2 and C3 algorithms [6], and a negative-binomial cusum were compared with the occurrence of events deemed by two experienced epidemiologists to be of potential public health importance using historical daily RRv disease notification data. RRv disease is among the most commonly notified diseases in Western Australia, with a total of 1099 cases notified in Western Australia (55.4 per 100,000 population) during 2004 [12]. RRv infections most frequently occur among middle-aged adults, and produce a range of clinical symptoms including fatigue and polyarthritis that typically last from months to years [13].

### Data

Historical daily RRv disease case notification data for Western Australia are not publicly available, and de-identified data were obtained from the National Notifiable Diseases Surveillance System (NNDSS) under agreement from the Department of Health Western Australia and the Australian Government Department of Health and Ageing. Data were available for analysis from the 1^st ^of January 1991 to the 10^th ^of September 2004 (5002 days). Cases of RRv disease are notified based on laboratory evidence of infection, however, this does not always imply a definitive diagnosis [14]. Due to under-presentation and the underuse of laboratory testing in endemic areas, notified rates of RRv disease are considered to underestimate true disease rates [13].

The use of the date of report as the reference date in this investigation is based on the rationale outlined by Farrington and coworkers [15], including its availability for all notified cases. The use of the report date will influence timeliness and sensitivity of outbreak detection as it incorporates additional variability between the onset of illness and the date of report [15], however, they are the most feasible data to monitor in prospective surveillance.

### Definition of outbreaks

As detailed historical records of prospectively-identified outbreaks of RRv disease in Western Australia were not available, retrospective expert epidemiological evaluation was used to identify the occurrence of events of epidemiological significance in the data. Daily time series data for the study period were reviewed by two independent epidemiologists. These highly experienced medical epidemiologists, although employed within the same university faculty for a year, both have a history of independent employment both within Australia and internationally. The time series graphs used to identify RRv disease outbreaks also displayed the approximate timing of any outbreaks that were documented in the Communicable Diseases Intelligence quarterly and annual surveillance reports [16], or were identified in a review of RRv in Australia [13].

Both epidemiologists independently identified events of epidemiological significance by labelling outbreak and non-outbreak periods based on a visual review of the data, information provided on documented outbreaks, and their knowledge of RRv disease epidemiology in Australia. Start and end dates of the retrospectively identified events were specified. A meeting between the researcher and the two epidemiologists was subsequently used to discuss any differences in the identification of events or their timing and produce an agreed standard.

There were two areas of difference in opinion among the epidemiologists. The first area related to minor differences in the timing of specified start and end dates for each event identified, and was resolved through a joint review of the data. The second area of difference was the labelling of hyper-endemic periods during August to December 1992 and February to June 1994 by one expert. The first epidemiologist indicated that the observed level of disease activity in practice could be of some concern; however, chose to concur with the second expert who believed that although the level of disease was different, it was difficult to label as an event of significance based on the level of notifications alone. No information was provided to expert reviewers on epidemiological linkages between cases or geographical distribution. Following the joint discussion, a total of 15 events of public health significance were identified, and these were considered to define 'outbreak' periods for the purposes of algorithm comparison.

### EARS algorithms

The EARS algorithms C1, C2 and C3 [6] were selected for evaluation due to their widespread use. We evaluated the performance of the EARS algorithms over a range of alarm levels. If the conventional alarm level (C1 = 2) is used, the C1 algorithm simplifies to the current value being greater than the baseline mean plus three standard deviations, which is based on the previous 7 days of data. The C2 algorithm differs from C1 in the use of a guard band of two days duration between the baseline and the current day being evaluated. The C3 algorithm also uses a two-day guard band, but calculates a partial sum for the last three days of the positive deviation of the current value from the mean [6]. The EARS algorithms are designed to signal when the cusum values exceed 2, which implies that the algorithm statistics have exceeded a level which is three standard deviations greater than the baseline mean.

The algorithms were run using the R statistical software based on the implementation of the algorithms in the EARS-X Excel software version [17]. To check for coding errors in the R implementation of EARS developed for this study, the outputs of both versions were compared based on six semi-synthetic datasets of 150 days in length with randomly inserted outbreaks of different magnitudes. No differences in performance between the excel and R versions of the algorithms were detected. The R code is provided in Additional file 1.

### Negative binomial cusum algorithms

A negative binomial cusum [2] was selected for testing due to the potential ability of this method to minimise false alarms associated with over-dispersed data. The negative binomial distribution can be described by two parameters, r and c, where over-dispersion is determined by the parameter c. The following two equations were used to determine the negative binomial parameter values based on the means (u) and variances (σ^2^) derived from selected baseline periods:

c_0 _= u/(σ^2 ^- u)

r = u^2^/(σ^2 ^- u)

If we consider r as given and monitor for changes in c from an in-control c_0 _to an out of control level c_1 _where c_1 _> c_0_, the decision interval cusum is given by [2]:

C_0 _= 0

C_n_^+ ^= max(0, C_n-1_^+ ^+ X_n _- k^+^)

where k^+ ^= r.ln [c_0_(1+c_1_)/c_1_(1+c_0_)]/ln [(1+c_0_)/(1+c_1_)]

The out of control level c_1 _was determined by calculating a fixed interval of two baseline standard deviations greater than the baseline-derived in control level c_0_, effectively setting the negative binomial cusum to detect a shift magnitude of two standard deviations above the baseline mean. To allow comparison with the EARS algorithms, the negative binomial cusum was evaluated with the baseline mean estimated using just 7 days of baseline data. Like the EARS algorithms C2 and C3, a guard band of 2 non-analysis days was used between the 7 days of data used to establish the baseline mean and variance, and the data for the current day. This guard band prevents the most recent data being included in the baseline estimates, which may be detrimental in the case of slowly increasing incidence being incorporated into the baseline. The negative binomial cusum was also tested using three additional baseline data period lengths: 14, 28 and 56 days. A zero-start method was evaluated, and cusums were not reset following alarms to allow exploration of the effects of different signal thresholds on performance. The R code implementation of the negative binomial cusum is provided in Additional File 1.

### Evaluation

To compare algorithm performance, empirical methods were used to determine the cut-off values for each algorithm that would produce equivalent false alarm rates. We investigate the performance of both algorithm types using false alarm rates of approximately 0.005 and 0.001, which implies that the cusums are expected on average to produce a false positive alarm approximately once every six months and three years respectively.

Although the cut-off value used to determine signalling of the cusums was varied to allow exploration of performance at different false alarm rates, the implementation of the C3 algorithm retains the threshold of 2 used to exclude large observed counts on the current or previous two days from the cusum total score, as implemented in the EARS-X software (see Additional File 1).

Performance comparisons were based on two main indicators: sensitivity, which describes the ability of the algorithm to detect outbreaks in the data at any time during each outbreak period; and timeliness, which describes the number of days from the beginning of each outbreak until the first signal for each outbreak. As sensitivity was defined as the signalling of the algorithm at any point during the defined outbreak periods, timeliness was determined only for outbreaks that were detected, and defined as the number of days between the first nominated outbreak day, and the day of the first signal during the outbreak period. Any signals that occurred during outbreak periods were considered valid and the first of these during each outbreak period was used to calculate the time to detection. Signals that occurred on non-outbreak days (days that were not considered to be of epidemiological significance), were considered false alarms, and the average proportion of false alarms was calculated as the total number of false alarms divided by the total number of non-outbreak days. A one per cent false alarm rate (0.01) is equivalent to an alarm occurring on average on one out of every one hundred non-outbreak days.

As the early detection of events of interest is important, timeliness was also summarised for each cusum as the proportion of outbreaks detected within the first 7 days. As performed for a previous evaluation of the EARS algorithms [18], we calculated a conditional average run length indicator based on the detection of an event within the first 7 days. To enable the generation of complete timeliness data for algorithm comparison, an additional outcome variable (adjusted timeliness) was also derived by allocating the total duration of each outbreak as the timeliness result if an outbreak was undetected.

The performance of each algorithm was also evaluated by comparing the area under the receiver operating characteristic (ROC) curve (AUC) [19]. The AUC calculations were performed using the trapz function of the CaTools package for the R statistical software [20]. To allow the consideration of both sensitivity and timeliness in AUC comparisons, a weighted AUC indicator (WtAUC) was also generated [19], which weights the contribution of the sensitivity component of the AUC indicator based on the proportion of time saved relative to a reference value. As no historical data were available to provide a reference value, a fixed value of 7 days was used. The weightings applied to the sensitivity data were calculated as (7-timeliness)/7, with a lower limit of zero. As such, outbreaks detected more than seven days after their commencement do not contribute to the weighted AUC indicator.

Formal statistical comparisons of sensitivity, timeliness, adjusted timeliness, AUC and weighted AUC for the EARS and negative binomial cusums were performed using Friedman Rank Sum Tests as implemented in the Stats package for the R software version 2.6.0 [21]. Selected multiple comparisons were performed to investigate difference in performance between the three EARS algorithms and the 7-day and 28-day negative binomial cusums using paired Wilcoxon Signed Rank tests. As these six analyses were conducted for each multiple comparisons procedure, the two-tailed p-value used to determine significance for the Wilcoxon Signed Rank tests was adjusted to reflect the repeated testing and set at 0.05/6, or 0.0083. Nonparametric methods were used due to the non-normailty of the data and small sample size available for analysis. This research was approved by the Human Research Ethics Committee of Curtin University of Technology.

## Results

We observed a good level of agreement between epidemiologists based on their independent evaluations, and good agreement between retrospective epidemiological review and larger documented outbreaks. Divergence between documented outbreaks at a national level and expert opinion only occurred for the smallest events identified by the epidemiologists, which are less likely to be of interest, or documented, at a national level.

### Descriptive statistics

Major outbreaks of RRv disease occurred approximately every four years between 1991 and 2004. Case notifications often remain elevated for 6 months following seasonal outbreaks, which generally occurred with a mid-point around the month of March. The multiple-year disease cycle was associated with large long term variation in baseline statistics due to the timing of the epidemic and inter-epidemic years. There was no clear trend towards increased case counts during the period analysed.

**Figure 1:**
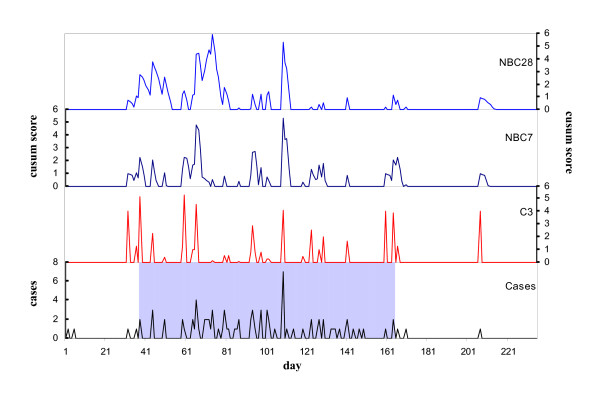
Ross River virus case notifications, expert-defined outbreak period (shaded) and cusum scores by day for the first outbreak dataset (days 1–235).

The mean of the daily count of RRv disease case notifications (1.53) was considerably lower than the variance (13.76), indicating that the data are overdispersed. When epidemiologically-defined outbreak periods were excluded (n = 2636), the mean RRv disease case count (0.26) remained lower than the variance (0.31). The mean number of cases reported per day for each expert-identified outbreak period varied between less than 1 case per day for smaller outbreaks in 1993 and 1995, to more than 8 cases per day for a large outbreak in 1996. The number of cases reported on the first day of identified outbreaks ranged between 2 and 4. The historical data are displayed in Figure 1 and Additional File 2, with the division of the data into 15 'outbreak' datasets based on equal bisection of each of the intervening non-outbreak periods.

On average, the use of longer baseline periods for the negative binomial cusum was associated with decreased variation in the out of control mean, and a higher average out of control mean. The negative binomial cusum out of control mean for the 7-day baseline model (4.56) was lower than the mean for the 14-day (4.64), 28-day (4.74) and 56-day (4.97) baseline models. The maximum value of the negative binomial cusum out of control mean for the 7-day baseline model (60.1) was higher than the maximum for the 14-day (55.2), 28-day (46.1) and 56-day (40.1) baseline models.

Large EARS and negative binomial cusum algorithm signals occurred on a number of days which were not classified as of epidemiological importance. Most commonly these false alarms occurred following isolated small-scale increases in notifications, or increases prior to a defined outbreak period. In July 2001 (Additional File 2: 11^th ^outbreak dataset, day 279) a large signal was issued by all algorithms on a day where an isolated spike of one then four cases were reported.

### Sensitivity

**Figure 2:**
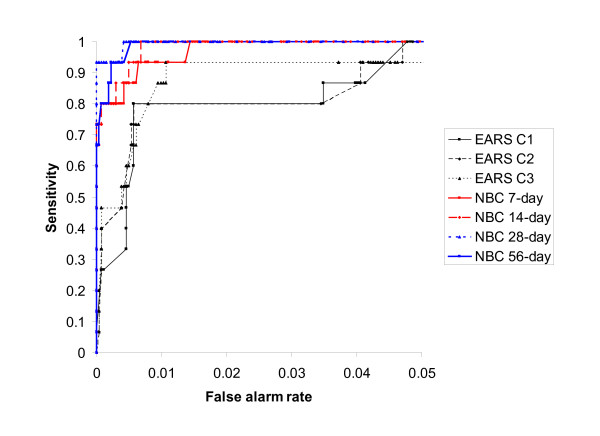
Sensitivity of Early Aberration Reporting System (EARS) and negative binomial cusum (NBC) algorithms according to false alarm rate.

Based on epidemiological opinion, the sensitivity of the EARS and negative binomial cusums were more similar at higher false alarm rates, as illustrated in Figure 2. At false alarm rates of less than 0.01, the negative binomial cusum had higher sensitivity than the EARS algorithms, and the sensitivity of the negative binomial cusum was generally higher when longer baseline periods were used. At a false alarm level of 0.005 the 28-day negative binomial cusum also had the highest sensitivity during the first 7 days of the outbreak (60%), followed by the C3 algorithm and the 7-day negative binomial cusum (both 40%) (Table 1). At a false alarm rate of approx 1 every 1000 days (0.001), the 7-day negative binomial cusum had the highest 7-day sensitivity (27%) (Table 2).

**Table 1:** Algorithm summary performance statistics for false alarm rates approximating 0.005^†^

Algorithm	false alarm rate	median (mean) sensitivity	Day 1 sensitivity	Day 2 sensitivity	Days 1–7 sensitivity	CARL	median (mean) timeliness^‡^	median (mean) adjusted timeliness^‡^
EARS C1	0.0049	1 (0.53)	0.13	0	0.13	1.0	18.5 (54.6)	34.0 (78.8)
EARS C2	0.0042	1 (0.53)	0.33	0	0.33	1.0	0.0 (24.1)	32.0 (71.1)
EARS C3	0.0049	1 (0.60)	0.40	0	0.40	1.0	0.0 (17.9)	32.0 (68.1)
NBC 7-day	0.0049	1 (0.87)	0.2	0.07	0.40	2.0	12.0 (16.3)	14.0 (18.5)
NBC 14-day	0.0049	1 (0.93)	0.2	0.07	0.33	1.6	9.5 (13.9)	11.0 (15.2)
NBC 28-day	0.0049	1 (1.00)	0.27	0.07	0.60	2.9	5.0 (8.5)	5.0 (8.5)
NBC 56-day	0.0042	1 (0.93)	0.13	0	0.27	3.0	14.5 (19.1)	14.0 (18.8)
p-value^¥^	-	0.0002	-	-	-	-	0.71	0.008

^†^Test threshold that produced a false alarm rate ≤ 0.005

^‡^Detection on the first outbreak day is equivalent to a timeliness of 0 days

^¥^Friedman rank sum test

CARL: Conditional Average Run Length – conditional on the detection of the outbreak during the first 7 days

EARS: Early Aberration Reporting System

NBC: Negative binomial cusum with an out of control state defined as 2 standard deviations greater than the mean

**Table 2:** Algorithm summary performance statistics for false alarm rates approximating 0.001^†^

Algorithm	false alarm rate	median (mean) sensitivity	Day 1 sensitivity	Day 2 sensitivity	Days 1–7 sensitivity	CARL	median (mean) timeliness^‡^	median (mean) adjusted timeliness^‡^
EARS C1	0.0008	0 (0.27)	0.13	0	0.13	1.0	36.5 (49.0)	123 (113.1)
EARS C2	0.0008	0 (0.40)	0.13	0	0.13	1.0	44.5 (52.7)	90 (96.0)
EARS C3	0.0008	0 (0.47)	0.13	0	0.13	1.0	51.0 (52.4)	73 (93.4)
NBC 7-day	0.0008	1 (0.80)	0.13	0.07	0.27	1.8	19.5 (32.3)	21 (31.1)
NBC 14-day	0.0008	1 (0.80)	0.07	0	0.13	2.0	33.5 (39.0)	32 (36.5)
NBC 28-day	0.0008	1 (0.93)	0.07	0	0.2	3.0	16.5 (26.3)	18 (26.8)
NBC 56-day	0.0008	1 (0.80)	0	0	0.13	5.0	19.0 (31.0)	20 (30.1)
p-value^¥^	-	<0.0001	-	-	-	-	0.91	0.0003

^†^Test threshold that produced a false alarm rate ≤ 0.001

^‡^Detection on the first outbreak day is equivalent to a timeliness of 0 days

^¥^Friedman rank sum test

CARL: Conditional Average Run Length – conditional on the detection of the outbreak during the first 7 days

EARS: Early Aberration Reporting System

NBC: Negative binomial cusum with an out of control state defined as 2 standard deviations greater than the mean

Comparison of the sensitivity of the seven cusum algorithms found significant differences at the 0.005 and 0.001 false alarm levels (Friedman χ^2 ^= 25.8, df = 6, p = 0.0002 and Friedman χ^2 ^= 39.2, df = 6, p < 0.0001 respectively, Tables 1 and 2). When the sensitivity of the EARS algorithms were individually compared with the sensitivity of the 7-day and 28-day negative binomial cusums at the 0.005 false alarm level, no comparisons were significant following adjustment for the number of comparisons performed, with p ≤ 0.02 for all comparisons with the 28-day negative binomial cusum, and p ≤ 0.07 for all comparisons with the 7-day negative binomial cusum. Multiple comparisons at the 0.001 false alarm level were significant for EARS C1 and the 7-day and 28-day negative binomial cusums, and C2 and the 28-day negative binomial cusum following adjustment for the number of comparisons performed (p = 0.006, p = 0.002 and p = 0.006 respectively), and p ≤ 0.04 for all remaining comparisons.

### Timeliness

The timeliness of the EARS algorithms exceeded that of the negative binomial cusum given that the outbreak was detected within the first 7 days (Tables 1 and 2). The negative binomial cusum was more likely than the EARS algorithms to signal between days 2 and 7. The negative binomial cusum also generally had a greater overall median time to detection at the 0.005 false alarm rate (Table 1), although the overall median time to detection for the negative binomial cusum was less than the EARS algorithms at the 0.001 false alarm level (Table 2). The 28-day negative binomial cusum was the most timely negative binomial cusum model tested based on all outbreaks detected at the 0.005 and 0.001 false alarm levels.

Comparison of the timeliness of the seven cusum algorithms found no significant difference at the 0.005 and 0.001 false alarm levels (Friedman χ^2 ^= 3.8, df = 6, p = 0.71 and Friedman χ^2 ^= 2.1, df = 6, p = 0.91 respectively, Tables 1 and 2). However, comparison of the adjusted timeliness of the seven cusum algorithms, which allocates the duration of the outbreak as the timeliness result when outbreaks are undetected, found a significant difference at the 0.005 and 0.001 levels (Friedman χ^2 ^= 17.3, p = 0.008 and Friedman χ^2 ^= 25.0, p = 0.0003 respectively, Tables 1 and 2). Multiple comparisons at both the 0.005 and 0.001 false alarm levels were only significant for EARS C1 and the 28-day negative binomial cusum following adjustment for the number of comparisons performed (p = 0.001 and p = 0.007 respectively), with p ≤ 0.05 for all remaining comparisons.

### AUC

Comparison of the AUC analyses for the seven cusum algorithms found a significant difference for the 0–1, 0–0.01, and the 0–0.005 false alarm ranges (Friedman χ^2 ^= 70.3, p < 0.0001, Friedman χ^2 ^= 70.6, p < 0.0001 and Friedman χ^2 ^= 61.9, p < 0.0001 respectively, Table 3). With the exception of the comparison of EARS C3 and the 7-day negative binomial cusum (p = 0.01 and p = 0.04 respectively), all multiple comparisons were significant after adjustment for repeated testing for the false alarm ranges 0–1 and 0–0.01 (all p ≤ 0.002). All multiple comparisons were significant after adjustment for repeated testing for the false alarm range 0–0.005 (all p ≤ 0.005).

**Table 3:** Descriptive statistics for summary performance characteristics for the EARS and NBC algorithms: receiver operating characteristic area under the curve (AUC) analyses

Algorithm	median (mean) AUC_0–1_	median (mean) AUC_0–0.01 _^†^	median (mean) AUC_0–0.005 _^‡^	median (mean) WtAUC_0–1_	median (mean) WtAUC_0–0.01 _^‡^
EARS C1	0.995 (0.989)	0.0011 (0.0019)	0.0004 (0.0015)	0.959 (0.968)	<0.0001 (0.0008)
EARS C2	0.996 (0.990)	0.0017 (0.0026)	0.0002 (0.0016)	0.994 (0.978)	0.0002 (0.0013)
EARS C3	0.996 (0.993)	0.0061 (0.0059)	0.0011 (0.0022)	0.991 (0.980)	0.0011 (0.0033)
NBC 7-day	1.0 (0.998)	0.0095 (0.0081)	0.0049 (0.0040)	0.990 (0.979)	<0.0001 (0.0032)
NBC 14-day	1.0 (0.999)	0.0099 (0.0088)	0.0049 (0.0040)	0.988 (0.981)	<0.0001 (0.0026)
NBC 28-day	1.0 (1.0)	0.0095 (0.0092)	0.0049 (0.0047)	0.990 (0.983)	0.0014 (0.0032)
NBC 56-day	1.0 (0.999)	0.0095 (0.0088)	0.0042 (0.0036)	0.977 (0.928)	<0.0001 (0.0018)
p-value^¥^	<0.0001	<0.0001	<0.0001	0.046	0.006

^†^Evaluated at a false alarm rate ≤ 0.01

^‡^Evaluated at a false alarm rate ≤ 0.005

^¥^Friedman rank sum test

WtAUC: Weighted AUC using a reference value of 7 days for valid detection

EARS: Early Aberration Reporting System

NBC: Negative binomial cusum with an out of control state defined as 2 standard deviations greater than the mean

Comparison of the weighted AUC analyses for the seven cusum algorithms found a significant difference for the 0–1 and 0–0.01 false alarm ranges (Friedman χ^2 ^= 12.8, p = 0.046 and Friedman χ^2 ^= 18.4, p = 0.006 respectively, Table 3). However, no multiple comparisons were significant after adjustment for repeated testing for the false alarm ranges 0–1 (all 0.17 ≤ p ≤ 0.85), and 0–0.01 (C1 all p ≤ 0.02, C2 all p ≤ 0.08, C3 all p ≤ 1.00). Comparison of the weighted AUC analyses for the seven cusum algorithms found no significant difference for the 0–0.005 false alarm range (Friedman χ^2 ^= 10.7, p = 0.10).

### Negative binomial cusum calibration

To investigate the influence of the negative binomial cusum settings on outbreak detection performance, we also evaluated the performance of the negative binomial cusum when the out of control state was defined as 3 standard deviations greater than the mean. Overall the timeliness and 7-day sensitivity of both the 2 and 3 standard deviation negative binomial cusum algorithms were comparable, with descriptive performance characteristics summarised in Table 4. At a false alarm level of 0.001 both the longer baseline 28-day and 56-day negative binomial cusums had an overall sensitivity of 80% based on a 3 standard deviation control limit.

**Table 4:** Summary performance statistics for false alarm rates approximating 0.005^† ^for the negative binomial cusum with an out of control state defined as 3 standard deviations greater than the mean

Algorithm	false alarm rate	mean sensitivity	Day 1 sensitivity	Day 2 sensitivity	Days 1–7 sensitivity	CARL	median timeliness^‡^
NBC 7-day	0.0049	0.80	0.2	0.07	0.33	1.6	13.0
NBC 14-day	0.0049	0.97	0.2	0.07	0.33	1.6	12.0
NBC 28-day	0.0049	1.00	0.27	0.07	0.60	3.0	6.0
NBC 56-day	0.0038	1.00	0.27	0.07	0.40	1.5	12.0

^†^Test threshold that produced a false alarm rate ≤ 0.00

^‡^Detection on the first outbreak day is equivalent to a timeliness of 0 days

CARL: Conditional Average Run Length – conditional on the detection of the outbreak during the first 7 days

NBC: Negative binomial cusum

## Discussion

This study explored the agreement between retrospective epidemiological opinion and the performance of two types of cusum algorithms for the detection of RRv disease outbreaks. The negative binomial cusum algorithm showed greater congruence with epidemiological opinion in terms of sensitivity, with the EARS algorithms having significantly lower AUC scores than the negative binomial cusum. However, when timeliness was incorporated into the AUC analyses, multiple comparisons between the EARS algorithms and the negative binomial cusum were no longer significant. This finding is associated with the ability of the EARS algorithms to signal an outbreak earlier if the outbreak was detected within the first seven days.

Our findings suggest that the use of a negative binomial distribution allows accommodation of the over-dispersion evident in disease notification data, and provides a lower rate of false alarms for a given sensitivity. However, improved sensitivity is associated with decreased early timeliness performance, particularly at higher false alarm rates. For the surveillance of RRv disease notification data, the improved sensitivity offered by the negative binomial cusum may outweigh the decrease in the timeliness of detection, as formal epidemiological review of the notification data does not routinely occur daily. Although the results of this study are based on a limited amount of historical data, they indicate the importance of examining the correspondence between the underlying model of the algorithm used and the data being monitored.

Despite the tendency of the EARS algorithms to have comparably high false alarm rates [8,22], previous studies have found cusums helpful for predicting and monitoring trends in influenza surveillance data [1,23]. The EARS system has been well-received as a method of automated monitoring as it is implemented using software likely to be familiar to epidemiologists, is relatively straightforward to set up, and has been favoured for its flexibility [1]. Selection of the most appropriate algorithms to implement in a specific surveillance context will depend on the surveillance objectives as well as disease-specific and operational considerations. These factors will determine the relative importance of the speed of detection, sensitivity and acceptable false alarm levels. In the case of cusums being applied to routinely collected disease notification data, the cost of false alarms may not be high if they are linked with procedures which facilitate efficient epidemiological review of relevant data to determine if further investigation or heightened monitoring is warranted.

Unlike a large-scale simulation study which found that algorithms, including the EARS C1, C2 and C3, did not reliably detect outbreaks of interest across a wide range of scenarios [24], our findings suggest that for RRv disease, a negative binomial cusum can reliably identify events of interest, although this method does not generally provide the same capability for the early detection of outbreaks as the EARS algorithms. The large nature of many RRv disease outbreaks, and the generally low level of baseline disease activity in the absence of recognised outbreaks provides conditions that have been associated with the more consistent detection of outbreaks using automated analysis methods [24]. Large-scale simulation studies are required to more fully define specific differences in the detection characteristics of the EARS and negative binomial cusum algorithms.

A limitation of this analysis in the context of evaluating the performance of cusum models is that cusums are optimal for the early detection of small sustained increases in the data being monitored [2], and the epidemiologist-nominated outbreak start dates may occur after the time of cusum signalling. As such, the methods used are likely to have resulted in the interpretation of several early outbreak signals as false alarms. An examination of false alarms occurring within 7 days of the commencement of the outbreaks revealed no consistent signalling of any single algorithm prior to the outbreak start dates, indicating that the specific start dates selected are unlikely to have biased the evaluation in favour of any particular algorithm.

The evaluation of outbreak detection algorithms in the absence of a well-accepted gold standard is challenging, and the small retrospective nature of this study is an important limitation of the current analysis. RRv disease notifications generally exhibit well defined outbreaks which are reasonably easily identified retrospectively; however, retrospective evaluation is unable to provide a definitive indicator of whether or when an outbreak has occurred, particularly for previously unrecognised or smaller-scale events. Furthermore, retrospective epidemiological judgements are not dependent on the case notification counts alone, but were made in the context of the epidemiologists' considerable history of experience in infectious disease epidemiology in Australia, and prior knowledge of the occurrence and epidemiological significance of previous outbreaks.

Despite the limitations of retrospective analysis, investigation of the correspondence between algorithm signals and epidemiological opinion can provide important information about the potential usefulness of outbreak detection algorithms in practice. Algorithms that are potentially useful for disease control can be expected to signal in a way that is consistent with retrospective epidemiological opinion. This investigation indicates that cusum algorithms can produce signals that are consistent with epidemiological opinion through the identification of a high proportion of RRv disease outbreaks at relatively low false alarm levels. The automated analysis of disease notification data has advantages associated with the consistency of data review, particularly given the large number of diseases under routine surveillance and the lack of resources for regular review of routinely collected data in the absence of other indications for heightened monitoring.

An advantage of the cusum implementations evaluated here is that signalling departures from the recent past removes the need to specify fixed parameters to describe the baseline level of disease activity, although there remains a need to define the amount of baseline data to be used for baseline estimation and the magnitude of shift of interest, which can both influence performance. Consistent with previous work [22], our findings demonstrate that the amount of historical data used to estimate the baseline level can have an important effect on algorithm performance. A 28-day baseline period for the negative binomial cusum generally produced improved overall performance with respect to sensitivity and timeliness in the case of RRv disease when compared with a 7-day baseline.

The challenge in designing algorithms to facilitate disease surveillance is to ensure that they quickly and consistently detect events of interest in the data. The evaluation of algorithms using historical data provides valuable information about performance in routine surveillance applications, contingent upon the outbreak definitions used. However, prospective studies are required to evaluate the value of automated surveillance systems in practice. Although this evaluation used a large proportion of the available historical data, performance evaluation was only based on the detection of 15 outbreaks. Further work is required to investigate the performance of negative binomial cusums more systematically using both large sample and prospective methods, and investigate the integration of this monitoring approach with other methods which may improve the sensitivity and timeliness of alerts and minimise the false alarms generated. Given the strong evidence of seasonal trends in RRv disease notifications, and previous studies indicating that time series approaches provide an effective method for automated surveillance [23,25], the investigation of these methods in future studies may also be worthwhile.

## Conclusion

We found reasonable agreement between negative binomial cusum performance and retrospective epidemiological opinion at low false alarm rates. The negative binomial cusum had a significantly greater ability to identify outbreaks of RRv disease that are considered epidemiologically significant than the EARS algorithms. However, when the timeliness of outbreak detection was considered in addition to sensitivity, there were no significant differences found between the performance of individual EARS and negative binomial cusum algorithms. Given that an outbreak was detected within the first seven days, the EARS algorithms were able to detect outbreaks more quickly when compared with the negative binomial cusum algorithms. Further work is required to explore the performance differences between the cusum models and determine if the application of these methods for automated surveillance are useful in practice.

## Competing interests

The authors declare that they have no competing interests.

## Authors' contributions

REW and AJP designed the study, REW conducted the study and drafted the manuscript, and SE, GW and BV were involved in revising the manuscript. All surviving authors read and approved the final manuscript.

## Pre-publication history

The pre-publication history for this paper can be accessed here:



## Supplementary Material

Additional file 1 R code for Early Aberration Reporting System (EARS) and negative binomial cusum (NBC) algorithms. Click here for file

Additional file 2 RRv case notifications, expert-defined outbreak period and cusum scores by day for outbreak datasets 2 to 15. Click here for file
