# Supplementary material for: Applying cusum-based methods for the detection of outbreaks of Ross River virus disease in Western Australia
Source: BMC Med Inform Decis Mak. 2008 Aug 13;8:37. doi: 10.1186/1472-6947-8-37 (PMC2542357; doi:10.1186/1472-6947-8-37)
Supplement: Additional file 1 — R code for Early Aberration Reporting System (EARS) and negative binomial cusum (NBC) algorithms. [file 1472-6947-8-37-S1.pdf]

Additional File 1: R code for Early Aberration Reporting System (EARS) and negative binomial cusum (NBC) algorithms

```
# CDC EARS CUSUM C1 C2 C3

# calculate mean and sd for the last 7-days and the last 7-days offset by 2 days

        u71[i] = mean(x[(i-7):(i-1)])

        sd71[i] = sd(x[(i-7):(i-1)])

        u93[i] = mean(x[(i-9):(i-3)])

        sd93[i] = sd(x[(i-9):(i-3)])

# adjust any sd=0 to = 0.2 (to avoid dividing by zero)

        sd71[sd71==0]= 0.2

        sd93[sd93==0] = 0.2

# calculate cusums C1 (S1), C2 (S2), C3 (S3)

        S1[i] = max(0, (x[i] - (u71[i] + sd71[i]))/sd71[i])

        S2[i] = max(0, (x[i] - (u93[i] + sd93[i]))/sd93[i])

# for C3 past S2 values are only included in S3 if they are not >2

        S3a[i]=ifelse(S2[i] > thr, 0, S2[i])

        S3b[i] = sum(S2[i], S3a[(i-1):(i-2)])

        S3[i]=ifelse(x[i] <= u93[i], 0, S3b[i])
```

```
## Negative binomial cusum with baseline based on the last 7 days with a 2 day lag

# calculate mean and variance

        u93[i] = mean(x[(i-9):(i-3)])

        var93[i] = var(x[(i-9):(i-3)])

# adjustment if mean and or var = 0

        if(u93[i]==0) {u93[i]<-0.1}

        if(var93[i]==0) {var93[i]<-0.105*u93[i]}

# adjustment if mean < = var

        if(u93[i]>=var93[i]) {var93[i]<- u93[i]*1.05}

# calculate NB parameters c and r (the in control c)

        c93[i] = 1/((var93[i]/u93[i])-1)

        r93[i] = u93[i]*c93[i]

# calculate out of control mean value as 2sd > observed mean

        ux93[i] = u93[i] + 2*((var93[i])^0.5)

# calculate out of control cx value based on out of control mean and fixed r

        cx93[i] = r93[i]/ux93[i]

# calculate k based on c, cx, r

        firstlog9[i] = (c93[i]*(1+cx93[i]))/(cx93[i]*(1+c93[i]))

        secondlog9[i] = (1+c93[i])/(1+cx93[i])

        k93[i]=r93[i]*(log(firstlog9[i]))/(log(secondlog9[i]))

# calculate negative binomial statistic C93 (positive deviations)

        C93[i] <- max(0,C93[i-1] + x[i] - k93[i])
```
