# Supplementary material for: Applying cusum-based methods for the detection of outbreaks of Ross River virus disease in Western Australia
Source: BMC Med Inform Decis Mak. 2008 Aug 13;8:37. doi: 10.1186/1472-6947-8-37 (PMC2542357; doi:10.1186/1472-6947-8-37)

Appendix 2 RRv case notifications, expert-defined outbreak period (shaded) and cusum scores by day for outbreak datasets 2 to 15.

Outbreak 2 (days 236-653)

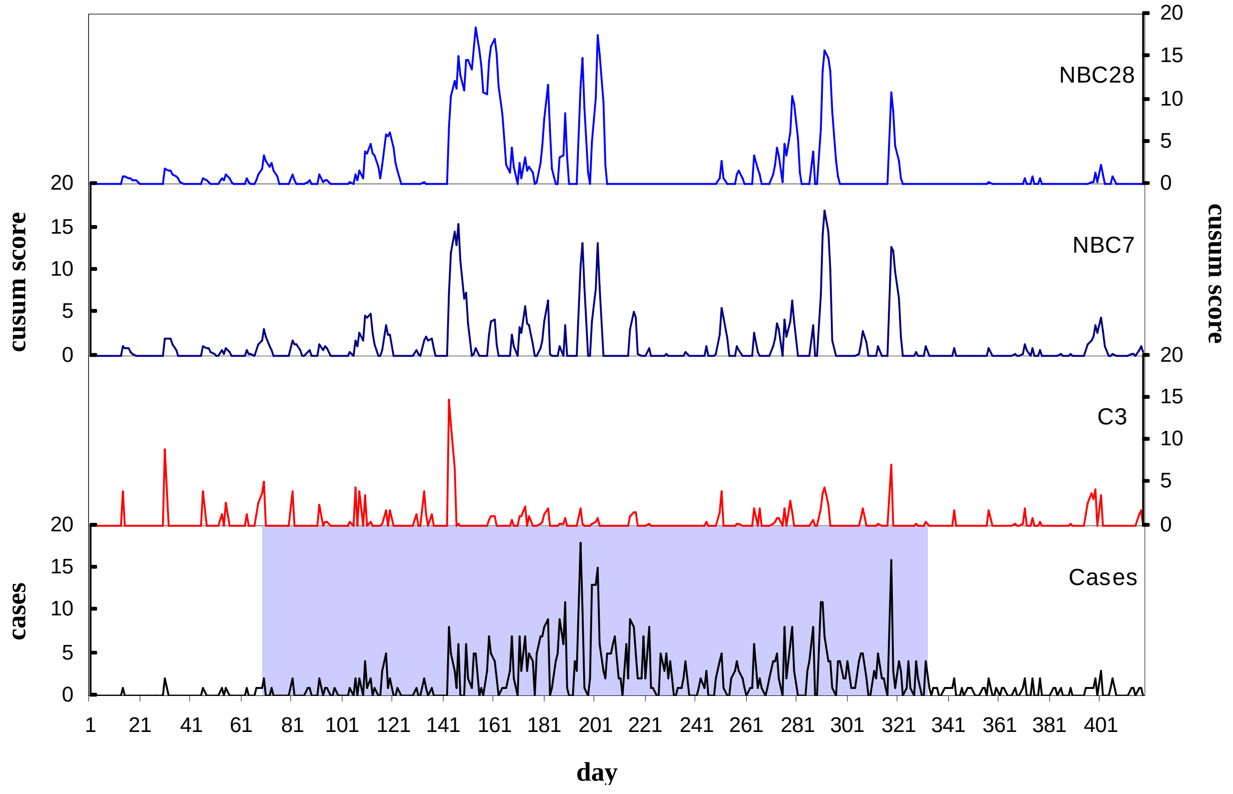

Outbreak 3 (days 654-891)

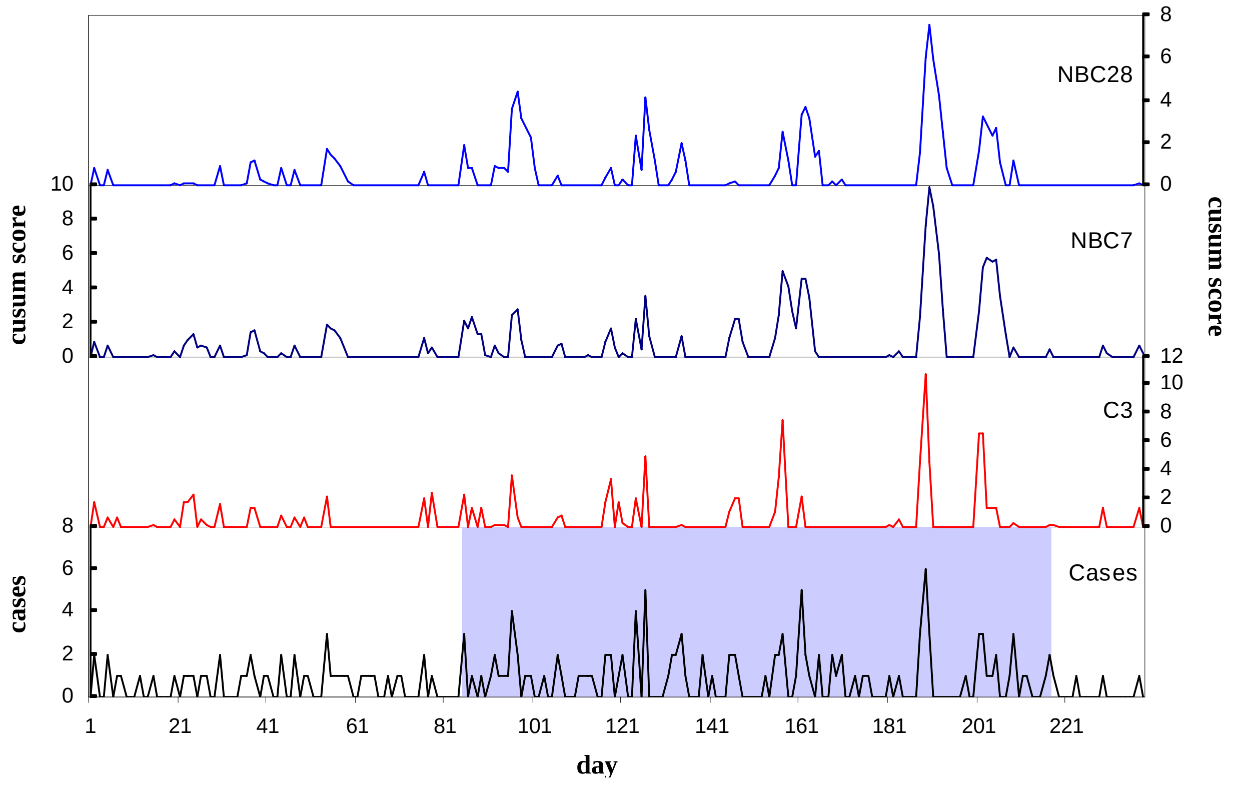

Outbreak 4 (days 892-1208)

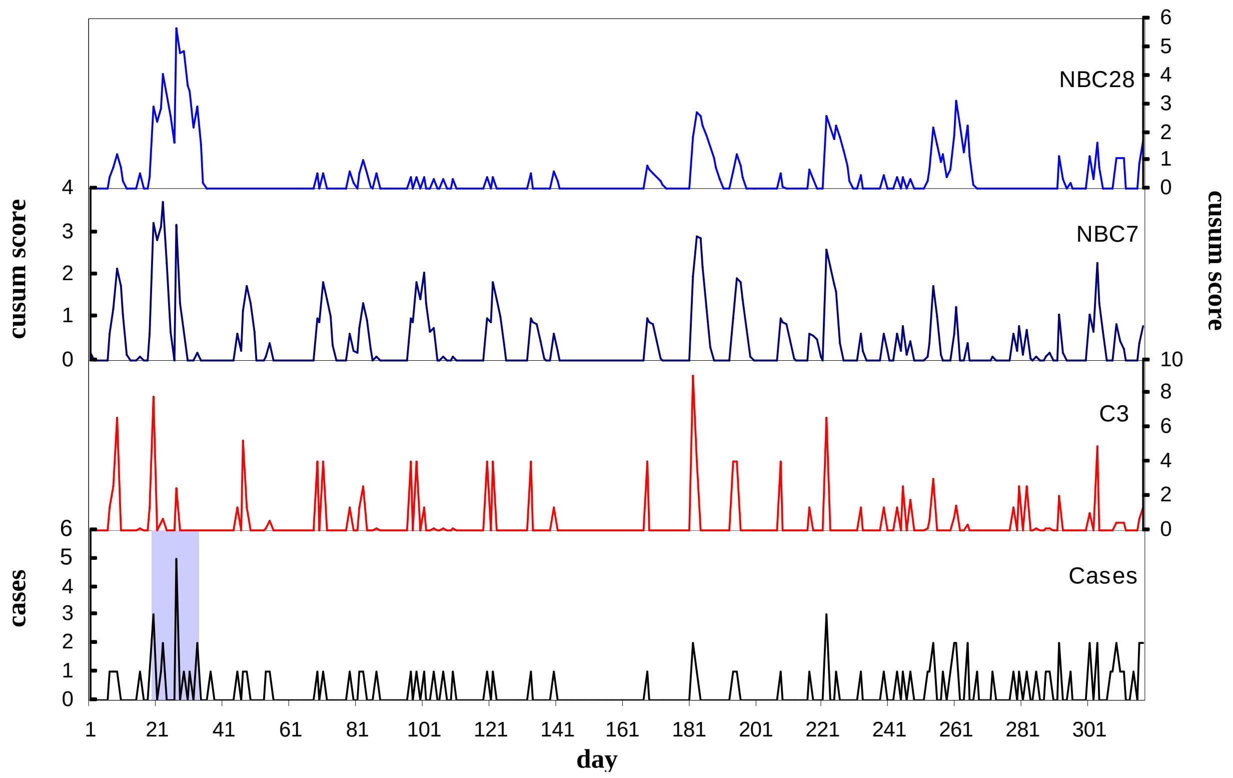

Outbreak 5 (days 1209-1749)

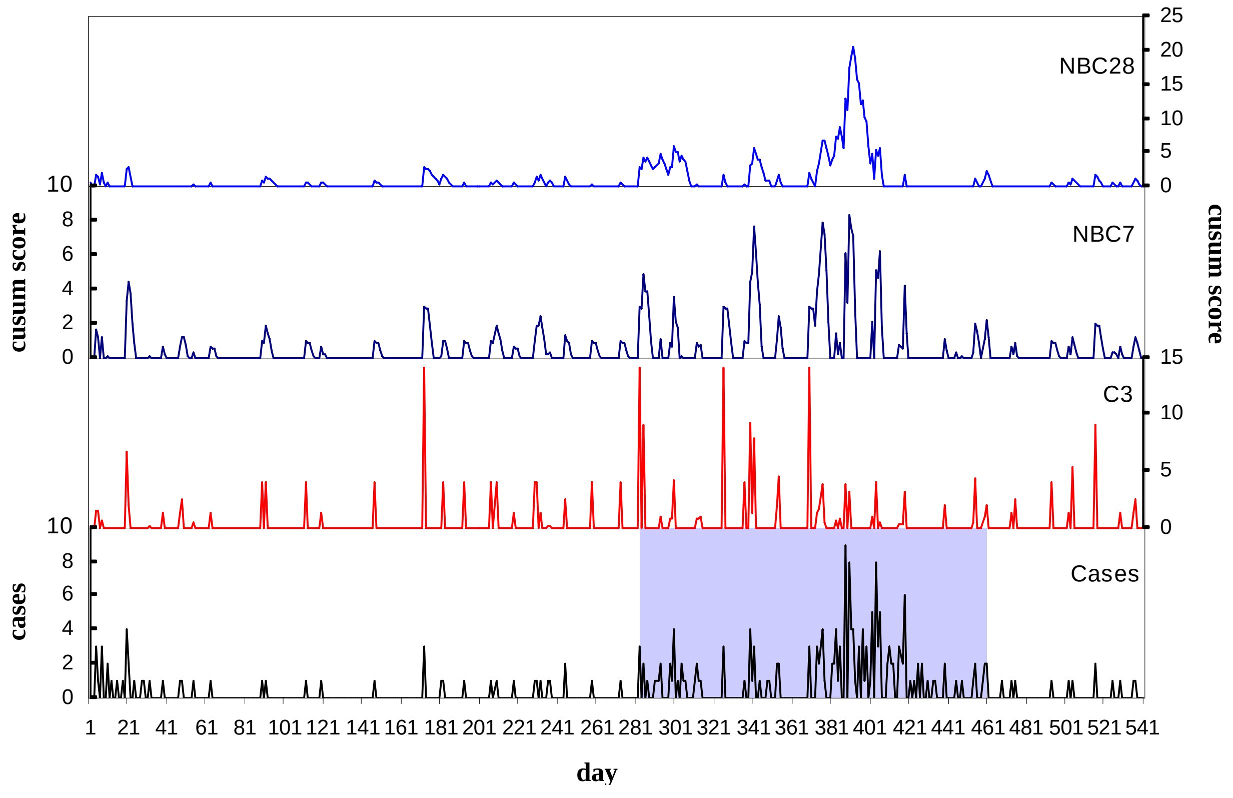

Outbreak 6 (days 1750-2066)

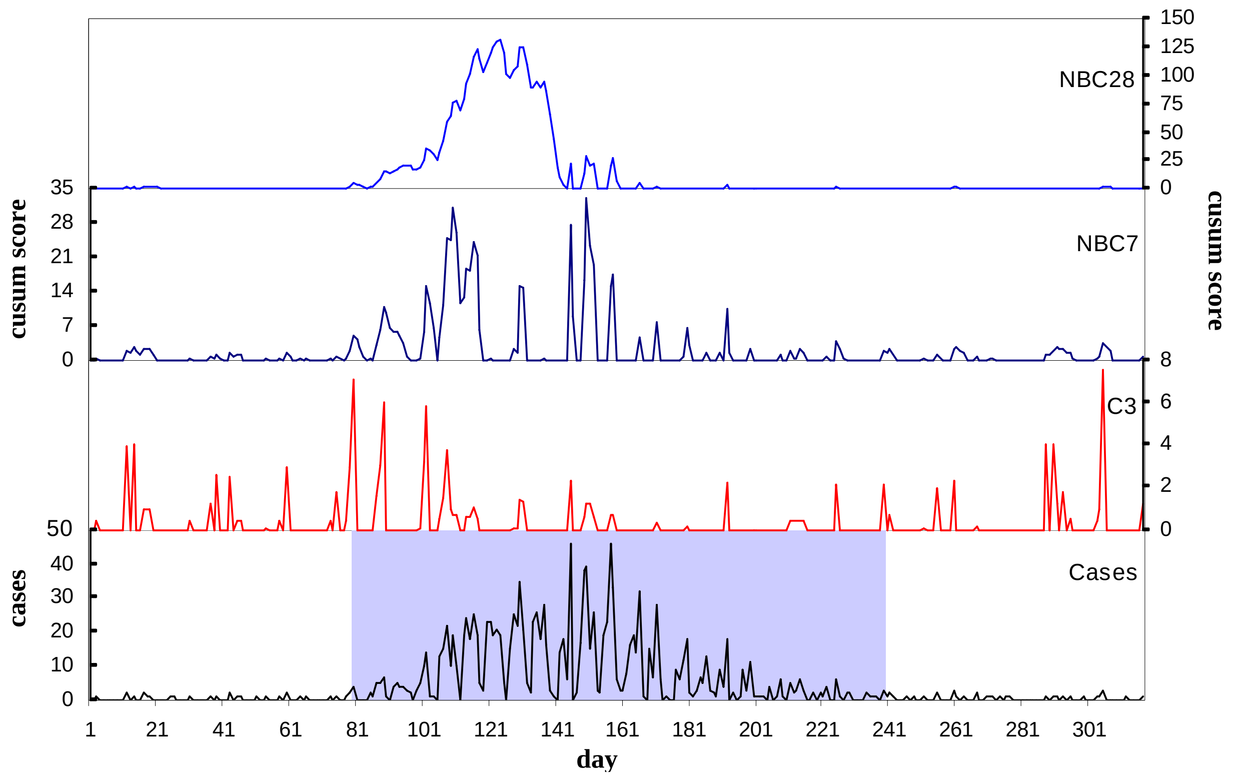

Outbreak 7 (days 2067-2492)

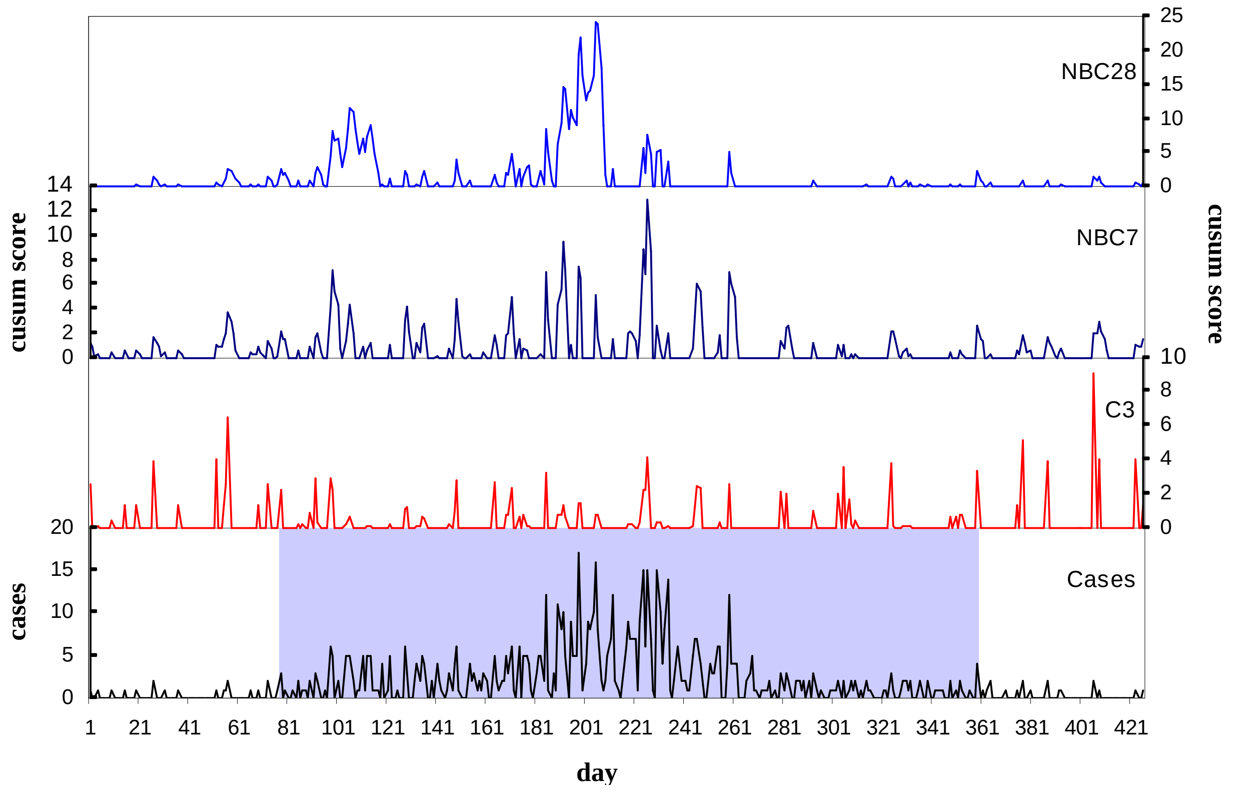

Outbreak 8 (days 2493-2781)

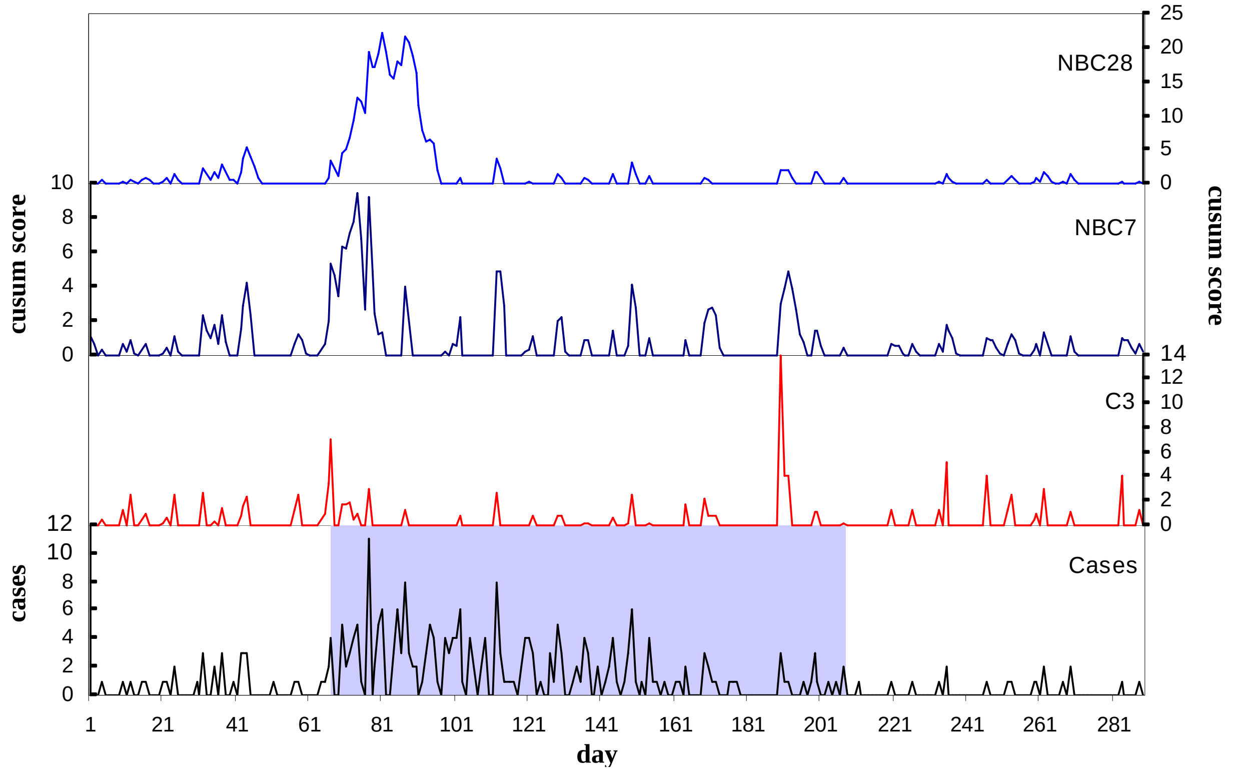

Outbreak 9 (days 2782-3188)

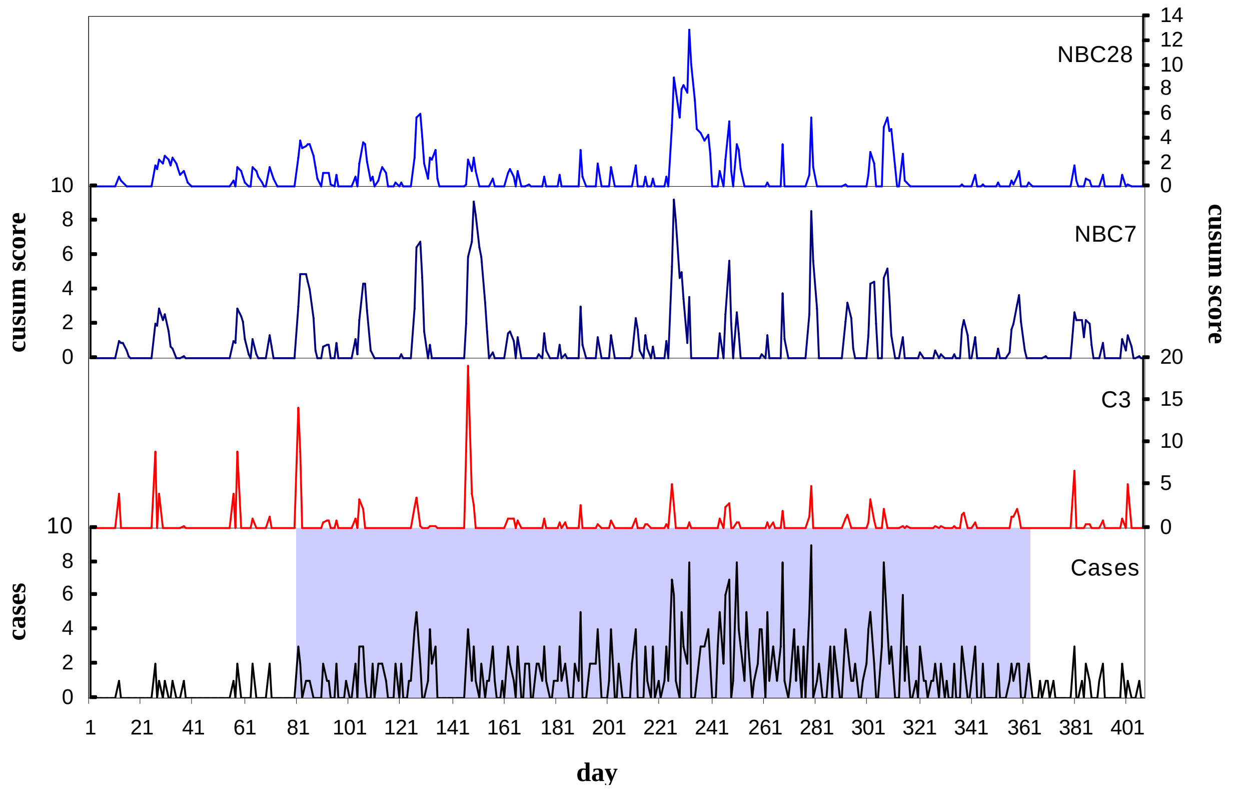

Outbreak 10 (days 3189-3581)

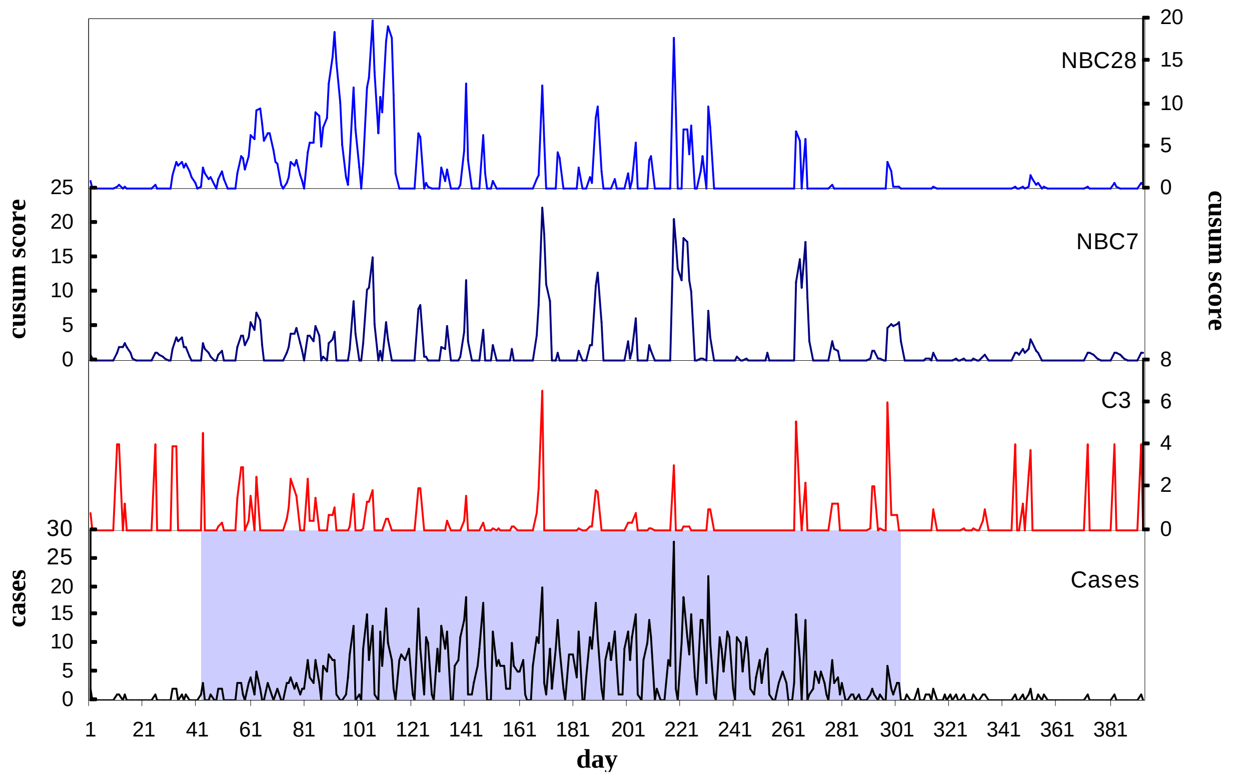

Outbreak 11 (days 3582-3917)

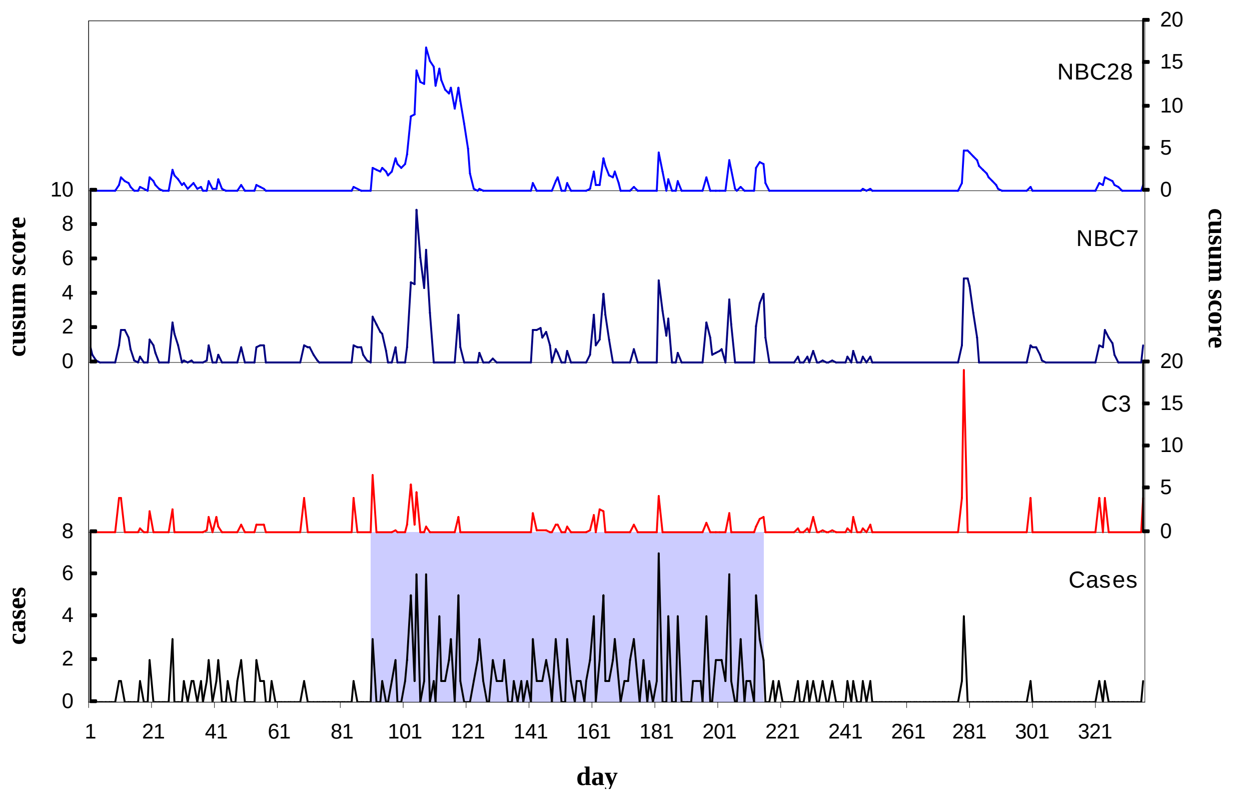

Outbreak 12 (days 3918-4085)

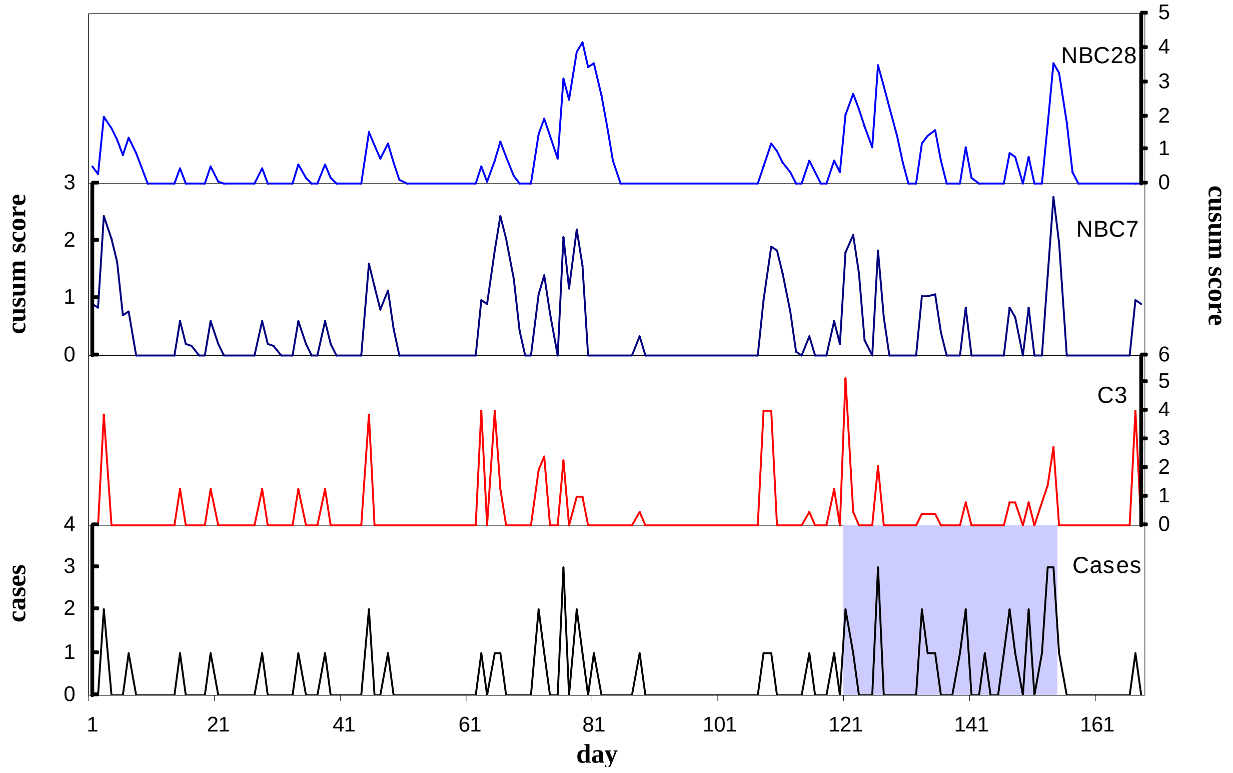

Outbreak 13 (days 4086-4297)

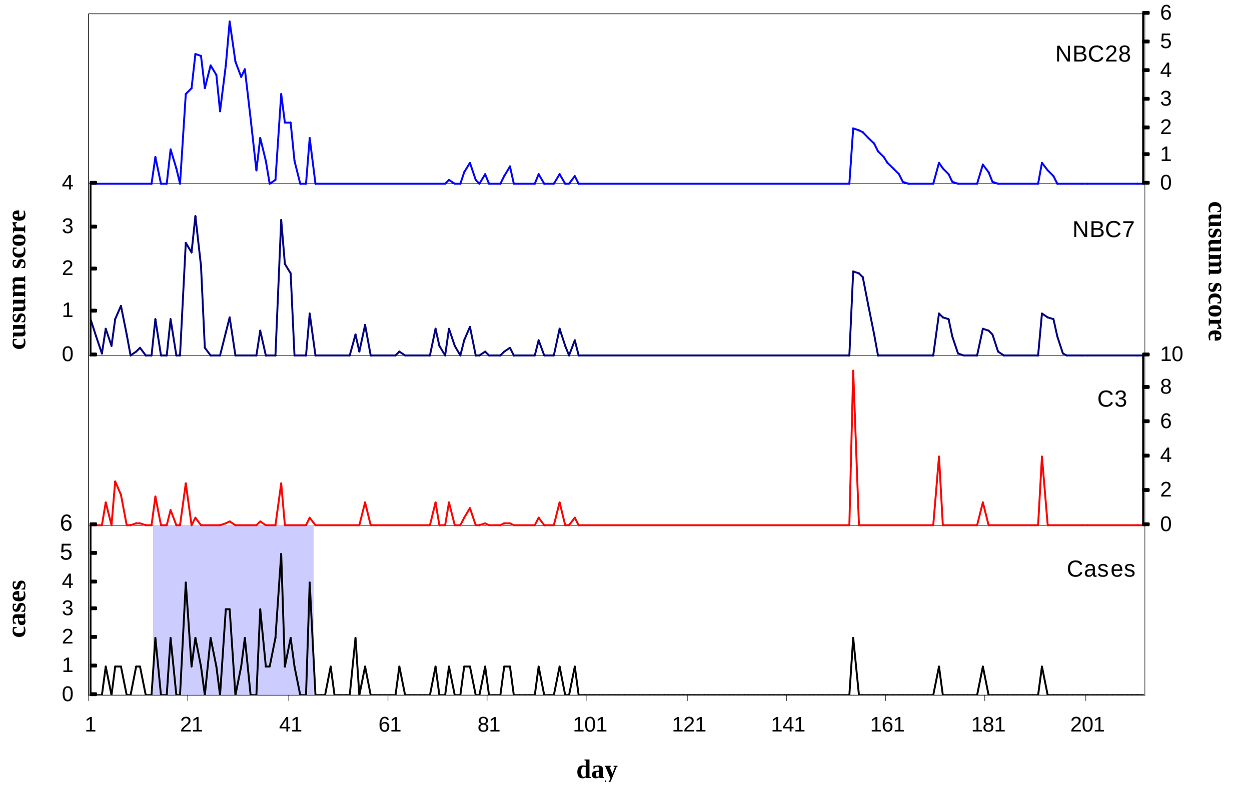

Outbreak 14 (days 4298-4626)

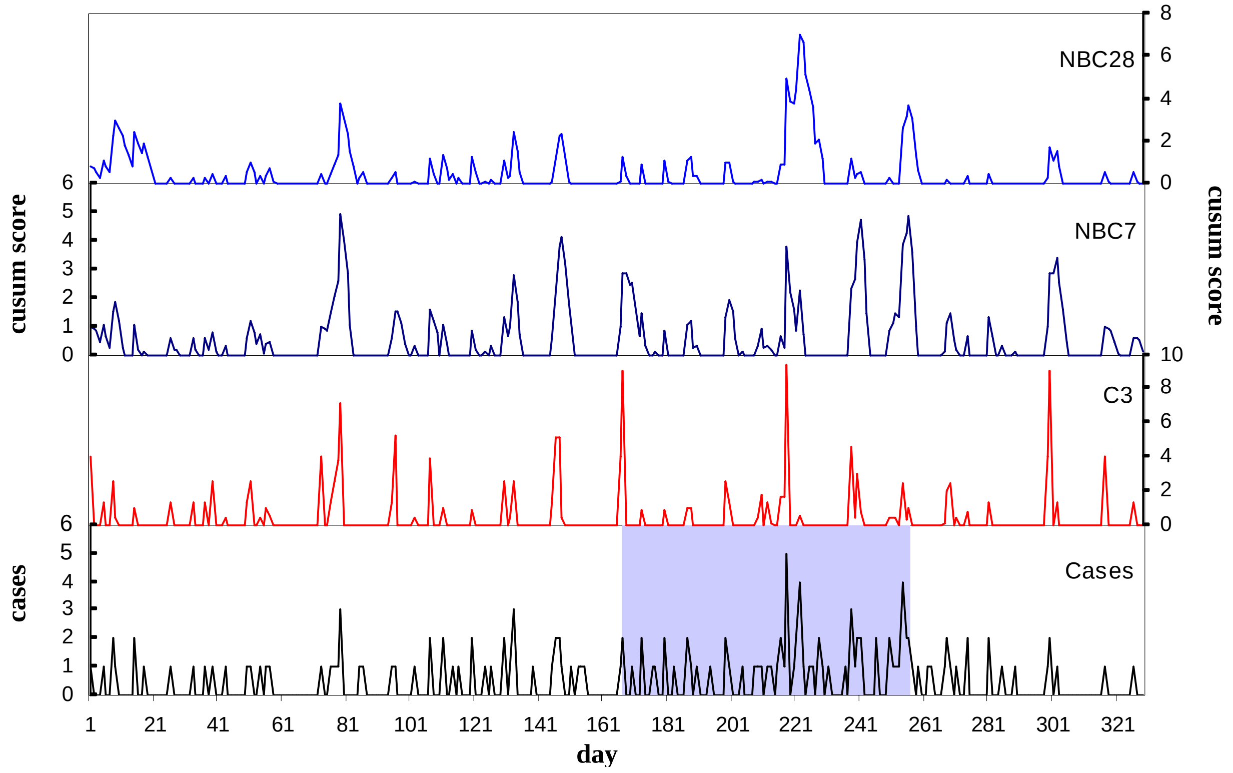

Outbreak 15 (days 4627-5002)

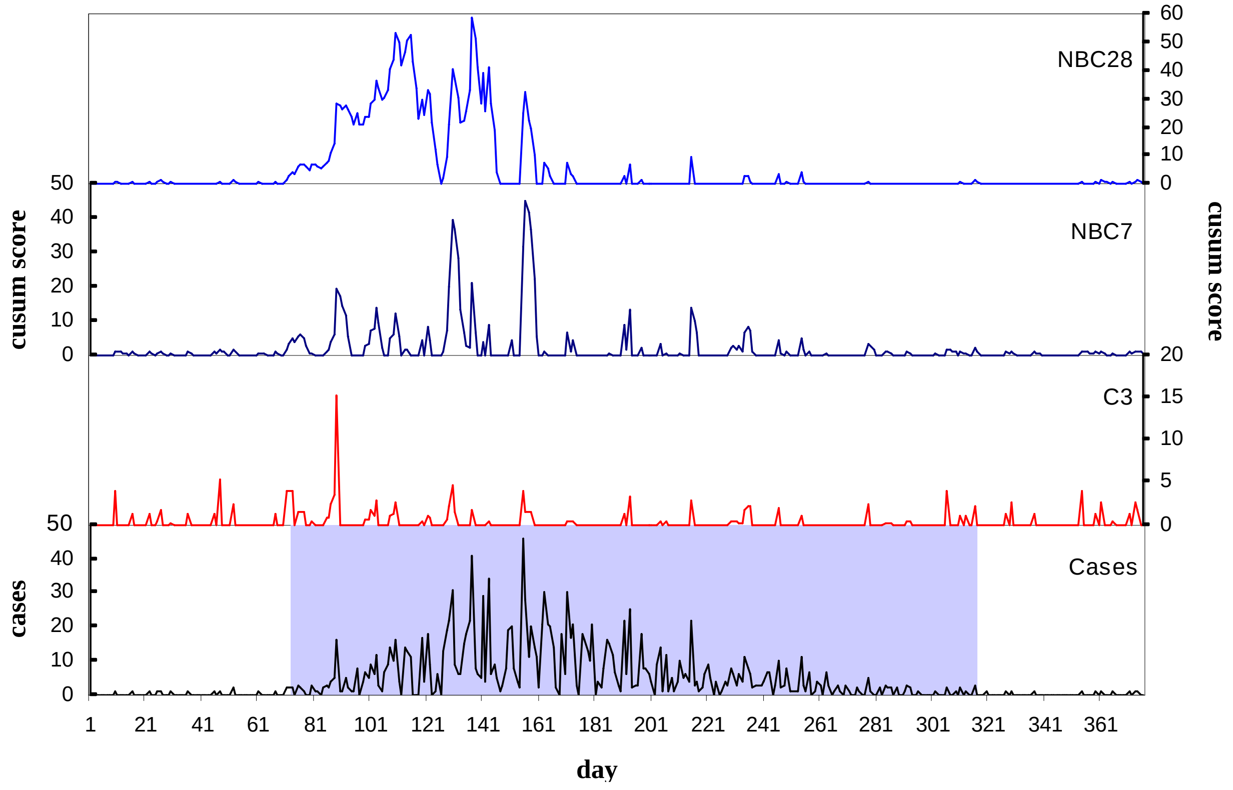

Supplement: Additional file 2 — RRv case notifications, expert-defined outbreak period and cusum scores by day for outbreak datasets 2 to 15. [file 1472-6947-8-37-S2.pdf]
